# The HBCU Climate and Heterosexual Black College Students: Gender Norms as Predictors of Sexual Deception

**DOI:** 10.1007/s12119-025-10387-4

**Published:** 2025-06-25

**Authors:** Jason M. Jones, Naomi M. Hall

**Affiliations:** Department of Psychological Sciences, Winston-Salem State University, Coltrane Hall, Winston-Salem, NC 27110 USA

**Keywords:** Sexual deception, Perceived mate availability, Black/African American, HBCU, Condom attitudes, Gender norms

## Abstract

Sexual deception involves dishonesty in interpersonal sexual relationships. Among heterosexual Black students at Historically Black Colleges and Universities (HBCUs), gender norms may contribute to these behaviors. Furthermore, gender ratio imbalances on HBCU campuses may influence these dynamics. While the literature on sexual deception at HBCUs is minimal, gender imbalances and media portrayals may impact interpersonal communication with sexual or romantic partners. This study examined the role of gender norms, condom attitudes, and perceived mate availability in sexual deception among Black HBCU students. A sample of 286 Black college students (ages 18–24, M = 20.37, SD = 1.70) from an HBCU in the southeastern United States completed surveys measuring sociodemographic information, conformity towards gender norms, sexual deception, perceived mate availability, and condom attitudes. Results indicated men believed the campus had an increased availability of mates, while women had more positive condom attitudes. In addition, self-serving deception was greater in casual or multiple casual relationships. Multiple regression revealed playboy and power over women norms in men predicted increased sexual deception, whereas fidelity and romantic relational norms predicted less sexual deception in women. Furthermore, the relationship between feminine norms and sexual deception varied based on relationship status. Findings highlight gender differences in sexual deception efforts. By examining how socially prescribed norms shape interpersonal behaviors, this study expands the literature on sexual deception and media influence, contributing to broader discussions on fostering healthier interpersonal dynamics in HBCU environments.

## Introduction

Sexual deception involves lying to have sex with a prospective or current partner (Marelich et al., 2008). This may occur through blatantly lying to achieve a desired outcome, acting in self-serving ways for personal gain, or avoiding confrontation with your partner. Blatant lies are told to achieve sexual pleasure and rewards. Self-serving deception is used for pleasure but includes the potential positive outcome (such as not being alone) with pleasure or reward. Lastly, avoiding confrontation is a deception to avoid adverse outcomes, such as not pleasing a partner or having sex to maintain the relationship.

At the societal level, literature, movies, and websites portray sexual deception as a tool for interpersonal gain (Marelich et al., 2008). Additionally, deception often accompanies presentations by each gender on how some believe men and women should behave, with both holding the potential for adverse personal and interpersonal outcomes (Jozkowski & Peterson, 2013; Marelich et al., 2008; Parent & Moradi, 2010). These outcomes range from rewards (i.e., sexual pleasure, money, maintaining companionship, coping with loneliness) to unfortunate circumstances such as unpleasant feelings, loneliness, and even sexually transmitted infections. Marelich et al. (2008) recommend educating young adults about deceptive practices to reduce these risks.

Sexual activity is common among young adult college students, and deception may be employed for various reasons (see Guthrie & Kunkel, 2013), irrespective of gender, sexual orientation, or relationship type. Some groups, such as Black college students, are often more upfront when deception occurs (see Easterling et al., 2019). Quirk et al. (2014) discovered that women endured more deception from their friends-with-benefits partners than men. However, Oswald et al. (2021) reported no significant difference in deception via blatant lying among undergraduate students. Research has shown that sexual deception in college students is grounded in belief systems such as endorsing gendered roles aligned with traditional sexual scripts (Jozkowski & Peterson, 2013). Despite this, literature on certain cultural groups has been scarce. This study aims to explore how young Black college students at a Historically Black College/University (HBCU) in heterosexual partnerships employ gendered norms in their sexual behaviors.

### The HBCU Climate

Few studies have examined sexual deception among Black college students, and even fewer focus on students at HBCUs. The HBCU environment, like any college/societal space, can influence the interpersonal dynamics of the men and women on campus (Johnson, 2017). Black cultural devices (i.e., religion, hip hop culture, media trends) can further inform more specific interpersonal norms that young Black men and women choose to adopt (i.e., monogamy, number of partners) with student peers (see Chandler et al., 2021; Johnson, 2017; Ward et al., 2020). Further, these influences can lead to interpersonal issues related to risky sexual behaviors (such as inconsistent condom use) that affect college students. These behaviors are further shaped by sociocultural factors unique to HBCU campuses (see Felder et al., 2019; Johnson, 2017; Reese et al., 2020).

HBCUs commonly experience gender ratio imbalances, where the enrollment ratio of Black women to men is at least two to one (Hall et al., 2014; Johnson, 2017). Black men often feel the availability of partners is sufficient and sometimes report multiple partnerships and a lack of openness in sexual history (see Johnson, 2017; Stackman et al., 2016). Conversely, Black women report insufficient partner availability, which influences sexual decisions, creates rivalries, and leads to power dynamics in relationships (Hall et al., 2014; Stackman et al., 2016). Further, women often choose to be less open about their past experiences when seeking partnerships due to fear of judgment by male partners (see Hall et al., 2014; Johnson, 2017). Researchers have not yet connected these dynamics to sexual deception. However, it is important to examine whether the adherence to societal norms within the HBCU climate, particularly the gender ratio imbalance, affects students' interpersonal communication and sexual behaviors.

### Theoretical Foundation

Social exchange theory (SET) (see Homans, 1961; Thibaut & Kelley, 1959) provides a foundation for understanding sexual deception. SET contends there is a cost–benefit analysis, where one partner may depend more on the other. Increased dependence indicates less power and greater reward for the person in the more powerful position (see Lawler & Thye, 2006). At HBCUs, gender ratio imbalances can create such power disparities. For heterosexual men, an abundance of mates can translate to the reward of multiple partners and reinforce the belief that commitment is unnecessary. In contrast, women may encounter fewer mate options, leading to the perception of reduced opportunities for love and intimacy, and potentially settling for a less desirable partner (Hall et al., 2014). Gender norms can influence how individuals accept their partner enacting a culturally expected norm socially, but not interpersonally, to maintain a relationship. This enactment could become an issue when desires are unmet. For example, a woman may choose to be committed to a man who maintains a certain social status as a “playboy.” This may not be a desirable mate preference; however, the social status of dating this well-known figure may outweigh his normed adherence. Furthermore, she may adhere to certain sexual behaviors that the man prefers, such as unprotected sex. In this scenario, the man can hold a position of power regarding their sexual activity and his perceived social status (see Hall et al., 2014). Alternatively, the woman can have specific requirements, such as condom use, but choose to adhere to certain norms at the cost of diminishing the man's reward to maintain the perception of his social status. Suppose the benefit of adhering to a gender norm comes with the cost of dishonesty. In such cases, there is potential for losing the reward and creating deeper interpersonal and individual issues, especially on HBCU campuses with gender ratio imbalances (see Hall et al., 2014; Johnson, 2017; Stackman et al., 2016). Findings from this study grant further utility to SET (see Homans, 1961; Thibaut & Kelley, 1959) through the potential understanding of the gender norms that accompany sexual communication and reward-seeking in Black college students. Furthermore, findings grant further understanding of which social norms (and condom attitudes) are enacted in reward-seeking by Black students at an HBCU campus comprised of a gender ratio imbalance (see Hall et al., 2014; Johnson, 2017; Stackman et al., 2016).

### Purpose of the Present Study

The choice to adhere to masculine or feminine norms on HBCU campuses with gender imbalances can lead to inconsistent condom use and interpersonal dishonesty (see Hall et al., 2014; Johnson, 2017). If these variables influence communication, this potential deception and dishonesty can create environmental risks on HBCU campuses and long-term adverse student outcomes (see Hall et al., 2014; Johnson, 2017; Younge et al., 2013). In addition, findings have implications for campus efforts to promote openness and transparency in sexual communication and interpersonal relations for young Black students and unpack cultural norms within the larger society. This study examines adherence to gender norms on an HBCU campus and its link to sexual deception in relationships. Predictors include conformity to gender norms, perceived mate availability, and condom attitudes. The following hypotheses will be tested: (1) Gender and relationship differences affect condom attitudes, perceived mate availability, and sexual deception in heterosexual Black HBCU students; (2) Conforming to gender norms, condom attitudes, and perceived mate availability predict sexual deception in men or women in heterosexual partnerships. This paper is a part of a more extensive mixed-method study with Black HBCU students.

## Method

### Participants

The study included 286 Black self-identified heterosexual college students (46.5% Male/ 53.5% female) attending an HBCU in the southeastern United States with a female-to-male ratio of approximately 2.5-to-1. The students’ ages ranged from 18 to 24 years (*M* = 20.37, *SD* = 1.70), with 21.0% freshman, 23.1% sophomore, 26.9% junior, 28.3% senior, and 0.7% in graduate education. Almost one-third (32.3%) identified as being in a committed relationship, while 4.5% identified as being committed/messing around, 11.9% as casual, 4.5% with multiple casual relationships, and 46.9% were not in any relationship. Participants were recruited on campus via Institutional Review Board (IRB)-approved fliers posted in common areas and face-to-face (f2f) contact. The fliers provided the eligibility criteria: (1) self-identify as Black or African American; (2) full-time college student between the ages of 18–24; (3) unmarried; (4) not knowingly pregnant or attempting to become pregnant; (5) not knowingly HIV positive; and (6) must have engaged in penetrative sexual activity (vaginal and/or anal) within the past 12 months. Students completed a brief pre-screening survey to determine eligibility.

Participants included in this study provided informed consent before completing the survey*.* Eligible students completed the quantitative assessments privately on a laptop equipped with Computer Assisted Survey Information Collection (CASIC) Builder. CASIC Builder is used for collecting data because computerized methods are highly successful in gathering sensitive or personal data from participants. The survey took about 45–60 min to complete. All participants were debriefed at the end of the survey. In addition, they were given contact information in case of follow-up questions or concerns. Finally, participants were provided with a gift card worth $25 for their time and effort.

### Measures

Several measures were used for this study. The study includes some Cronbach’s alphas less than the standard 0.7; however, scores between 0.60 and 0.69 are recognized as above satisfactory and permissible (Taber, 2018). Further, if the items are interpretable by the participants sampled, it is possible to use instruments without high levels of alpha (Taber, 2018). Table 1 presents an overview of the measures used in the study, including their Cronbach’s alpha, number of items, an example prompt, and the response scales for the following:

1. Conformity to Masculine Norms Inventory (CMNI) (Parent & Moradi, 2009)—An abbreviated version where male participants reported their personal actions, feelings, and beliefs into the listed five categories. Higher scores indicate greater conformity to norms.
2. Conformity to Feminine Norms Inventory (CFNI) (Parent & Moradi, 2010)—An abbreviated version where female participants reported their personal actions, feelings, and beliefs into the listed four categories. Higher scores indicate greater conformity to norms.
3. The Sexual Deception Scale (Marelich et al., 2008)—Participants respond to prompts on whether they have deceived a romantic partner. Higher scores indicate increased sexual deception.
4. The Perceived Mate Availability scale (Hall et al., 2014)—Participants report if they believe there to be an availability of romantic partners (mates) on their campus. Higher scores indicate less perceived availability of mates on campus.
5. Condom Use Attitudes Scale (Boyer et al., 2008)—Participants report their feelings towards condom use in sexual intercourse. Higher scores indicate more positive attitudes toward using condoms.

**Table 1:** Measure Descriptions, Examples, and Reliability

Measures	Cronbach’s alpha (α)	Number of items	Example item	Response scale
*Conformity to Masculine Norms Inventory* (Parent & Moradi, 2009)	0.87	25		1 = Strongly Disagree to 4 = Strongly Agree
Emotional Control	0.89	6	*"I never share my feelings."*	
Playboy	0.73	4	*"I would feel good if I had many sexual partners."*	
Power Over Women	0.76	4	*"Things tend to be better when men are in charge."*	
Self-Reliance	0.67	6	*"I never ask for help."*	
Heterosexual Self-Presentation	0.87	6	*"Being thought of as gay is not a bad thing."*	
*Conformity to Feminine Norms Inventory* (Parent & Moradi, 2010)	0.71	20		1 = Strongly Disagree to 4 = Strongly Agree
Appearance	0.70	5	*"I regularly wear makeup."*	
Fidelity	0.75	5	*"I would feel guilty if I had a one-night stand."*	
Relational	0.66	5	*"I make it a point to get together with my friends regularly."*	
Romantic Relationship	0.69	5	*"Being in a romantic relationship is important."*	
*Sexual Deception Scale* (Marelich et al., 2008)	0.83	19		1 = Yes, 2 = No
Blatant Lying	0.75	6	*"Told someone they’d be your boyfriend/girlfriend just so they would have sex with you?"*	
Self-Serving	0.73	3	*"Had sex with someone so you would have someone to sleep next to?"*	
Avoiding Confrontation	0.63	6	*"Had sex with someone so they wouldn’t break up with you?"*	
*Perceived Mate Availability* (Hall et al., 2014)	0.83	6	*"There is a shortage of eligible Black men/women for me to date."*	1 = Strongly Disagree to 6 = Strongly Agree
*Condom Use Attitudes* (Boyer et al., 2008)	0.76	5	*"Condoms decrease the feeling during sex."*	1 = Strongly Agree to 5 = Strongly Disagree

### Data Analysis

All data were exported and analyzed using the Statistical Package for the Social Sciences (SPSS; v.28) and included:

1. Pearson’s correlation to examine the relationships between any of the three predictor variables and sexual deception.
2. Independent samples t-test to assess any gender differences for the mentioned variables.
3. Analysis of variance (ANOVA) with post hoc Tukey test was conducted to evaluate the adherence to norms, condom attitudes, and perceived mate availability based on relationship status. This analysis aimed to understand how these factors were influenced by a gender ratio imbalance.
4. Multiple regression was used to determine how gender norms, condom attitudes, and perceived mate availability are associated with sexual deception. It also assessed these associations based on relationship type.

## Results

### Preliminary Analyses

Pearson’s correlation analyses were conducted to examine the relationship between key constructs and assess whether demographic factors were related to these constructs. Findings indicated that age was positively correlated to overall sexual deception *r*(284) = .26, *p* < .001, as well as its subtypes: blatant lying *r*(284) = .19, *p* < .01; self-serving deception* r*(284) = 0.16, *p* < .01; and deception to avoid confrontation *r*(284) = .25, *p* < .001. These results suggest that as students get older, they may be more likely to engage in sexually deceptive behaviors.

### Gender and Relationship Differences

#### Gender Differences

To test the first hypothesis, gender differences were examined across three variables: condom attitudes, perceived mate availability, and sexual deception. Men perceived significantly greater mate availability compared to women, *t*(284) = − 2.19, *p* < .05. However, women held significantly more positive attitudes towards condom use than men, *t*(284) =  − 4.65, *p* < .001. Men reported significantly greater use of deception to avoid confrontation compared to women *t*(284) = 3.22, *p* < .001.

#### Relationship Status Differences

To further assess the first hypothesis, differences based on relationship status were analyzed using analysis of variance.

*Perceived Mate Availability*. There was a significant main effect of relationship status with perceived mate availability *F*(4, 281) = 80.00, *p* < .001. Post hoc Tukey analysis revealed that students in casual or no relationships perceived significantly lower mate availability compared to those in committed relationships.

*Condom Attitudes.* A significant main effect of condom attitudes and relationship status was found *F*(4, 281) = 4.43, *p* < .01. Post hoc Tukey analysis revealed that those not in a relationship had significantly more positive condom attitudes than those in a committed relationship and those in a committed relationship that mess around.

*Self-Serving Sexual Deception*. There was a significant main effect with self-serving sexual deception and relationship status *F*(4, 281) = 4.57 *p* < .001. Post hoc Tukey analysis showed that students in casual and multiple casual relationships reported significantly more self-serving deception than those in committed relationships.

#### Relationship Type and Associated Variables

To further explore relationship differences, multiple regression analyses were conducted to determine if gender norms, condom attitudes, and perceived mate availability were significantly associated with sexual deception in each relationship type (see Table 2).

**Table 2:** Results of Multiple Regression Analysis in Norms and Sexual Deception Subscales by Relationship Status

Type of Sexual Deception	*t*	*p*	β	*F*	*df*	*p*	adj. R^2^
*Sexual Deception (Full/Casual Relationship)*							
Overall Model				4.67	3, 18	.01	.34
Condom Attitudes	− 2.82	.01	− .54				
Feminine Norms	2.93	.01	.54				
Mate Availability	.97	.35	.20				
*Sexual Deception (Full/No Relationship)*							
Overall Model				2.82	3, 62	.05	.08
Condom Attitudes	− 1.92	.06	− .24				
Feminine Norms	− 2.57	.01	− .32				
Mate Availability	− .38	.70	− .05				
*SD/Blatant Lying (Committed Relationship)*							
Overall Model				3.00	6, 46	.02	.19
Mate Availability	.19	.85	.03				
Condom Attitudes	.92	.36	.12				
Appearance	− .69	.49	− .09				
Fidelity	− 3.84	.00	− .50				
Relational	− .36	.72	− .05				
Romantic Relational	1.84	.07	.24				
*SD/Blatant Lying (Casual Relationship)*							
Overall model				3.28	6, 15	.03	.39
Mate Availability	1.63	.12	.32				
Condom Attitudes	− .29	.78	− .06				
Appearance	2.69	.02	.53				
Fidelity	− .51	.62	− .09				
Relational	1.34	.20	.24				
Romantic Relational	2.16	.05	.40				
*SD/Blatant Lying (No Relationship)*							
Overall Model				2.66	6, 59	.02	.13
Mate Availability	− 1.12	.27	− .14				
Condom Attitudes	− 2.41	.02	− .29				
Appearance	− 1.34	.19	− .19				
Fidelity	− 3.10	.00	− .39				
Relational	− .76	.45	− .09				
Romantic Relational	− .78	.44	− .11				
*SD/Self-Serving (Casual Relationship)*							
Overall Model				2.93	6, 15	.04	.36
Mate Availability	1.17	.26	.24				
Condom Attitudes	− 3.25	.01	− .63				
Appearance	2.54	.02	.51				
Fidelity	.12	.91	.02				
Relational	− .97	.35	− .18				
Romantic Relational	.43	.67	.08				
*SD/Avoiding Confrontation (Committed Relationship)*							
Overall Model				3.98	6, 46	.00	.26
Mate Availability	.74	.46	.09				
Condom Attitudes	− .16	.88	− .02				
Appearance	− .28	.78	− .04				
Fidelity	− 2.14	.04	− .27				
Relational	− 1.37	.18	− .18				
Romantic Relational	4.29	.00	.53				

*Women in Casual Relationships*. A model including overall feminine norms, condom attitudes, and mate availability significantly predicted sexual deception *F*(3, 18) = 4.67, *p* < .05. However, only increased feminine norms and negative condom attitudes were significant predictors.

*Women in No Relationships (Single Women)*. The same model showed that decreased adherence to feminine norms was significantly associated with increased deception *F*(3, 62) = 2.82, *p* < .05.

*Men*. Masculine norms, condom attitudes, and perceived mate availability did not significantly predict sexual deception based on relationship type.

#### Relationship Status and Feminine Norms

To determine if the feminine norms subscales, condom attitudes, and perceived mate availability were associated with sexual deception subscales based on relationship type, multiple regression analyses were conducted (see Table 2).

*Women in Committed Relationships*. The model significantly predicted deception via blatant lying *F*(6, 46) = 3.00, *p* < .05; however, increased fidelity was the only significant inverse predictor.

*Women in Casual Relationships*. The same model predicted blatant lying *F*(6, 15) = 3.28, *p* < .05, with increased appearance and romantic relational norms as significant positive predictors. In addition, increased appearance norms and negative condom attitudes were associated with greater self-serving deception *F*(6, 15) = 2.93, *p* < .05.

*Women in No Relationship*. The same model predicted blatant lying for women in no relationship *F*(6, 59) = 2.66, *p* < .05, with fewer fidelity norms and negative condom attitudes being significant inverse predictors.

*Women in Committed Relationships (Avoiding Confrontation)*. The model significantly predicted increased avoiding confrontation *F*(6, 46) = 3.98, *p* < .001, with increased romantic relational norms and decreased fidelity norms as significant predictors.

*Men*. The masculine norms subscales, condom attitudes, and perceived mate availability did not predict each sexual deception subscale based on relationship type.

### Gender Norms and Sexual Deception

#### Overall Norms

To examine the second hypothesis, multiple regression analyses were conducted to determine whether gender norms (masculine or feminine), condom attitudes, and perceived mate availability were associated with sexual deception.

*Women*. The model was not associated with sexual deception *F*(3, 149) = 1.56, *p* = .20.

*Men*. The model showed a significant positive association with sexual deception overall *F*(3, 129) = 3.27, *p* < .05; however, total masculine norms were the only significant predictor (see Table 3 & 4).

**Table 3:** Results of Multiple Regression Analysis in Masculine Norms and Sexual Deception Subscales

Type of Sexual Deception	*t*	*p*	β	*F*	*df*	*p*	adj. R^2^
*Sexual Deception (full)*							
Overall Model				3.27	3, 129	.02	.05
Masculine Norms (full)	2.22	.03	.20				
Condom Attitudes	− 1.78	.08	− .15				
Mate Availability	.31	.76	.03				
*Blatant Lying*							
Overall Model				4.19	7, 125	.00	.15
Mate Availability	− .34	.74	− .03				
Condom Attitudes	− 1.12	.27	− .09				
Emotional Control	− 1.66	.10	− .17				
Heterosexual Self-Presentation	.71	.48	.06				
Playboy	2.47	.02	.22				
Power Over Women	2.18	.03	.20				
Self-Reliance	1.30	.20	.14				
*Self-Serving*							
Overall Model				2.87	7, 125	.01	.09
Mate Availability	.77	.44	.07				
Condom Attitudes	− 1.32	.19	− .11				
Emotional Control	− 1.19	.24	− .12				
Heterosexual Self-Presentation	1.55	.12	.14				
Playboy	2.67	.01	.25				
Power Over Women	.43	.67	.04				
Self-Reliance	− .81	.42	− .09				
*Avoiding Confrontation*							
Overall Model				2.28	7, 125	.03	.06
Mate Availability	.04	.97	.00				
Condom Attitudes	− .04	.97	− .00				
Emotional Control	− 1.82	.07	− .19				
Heterosexual Self-Presentation	1.42	.16	.13				
Playboy	1.35	.18	.13				
Power Over Women	1.78	.08	.17				
Self-Reliance	.29	.77	.03

**Table 4:** Results of Multiple Regression Analysis in Feminine Norms and Sexual Deception Subscales

Type of Sexual Deception	*t*	*p*	β	*F*	*df*	*p*	adj. R^2^
*Sexual Deception (Full)*							
Overall Model				1.56	3, 149	.20	.01
Condom Attitudes	− 1.91	.06	− .16				
Feminine Norms	− 1.10	.27	− .09				
Mate Availability	− .07	.95	− .01				
*Blatant Lying*							
Overall Model				3.44	6, 146	.00	.09
Mate Availability	− .66	.51	− .05				
Condom Attitudes	− .99	.32	− .08				
Romantic Relational	1.08	.28	.09				
Relational	.45	.65	.04				
Fidelity	− 4.24	.00	− .34				
Appearance	.23	.82	− .02				
*Self-Serving*							
Overall Model				3.71	6, 146	.00	.10
Mate Availability	− .70	.49	− .06				
Condom Attitudes	− 2.37	.02	− .19				
Romantic Relational	.51	.61	.04				
Relational	− .66	.51	− .05				
Fidelity	− 3.82	.00	− .31				
Appearance	.52	.60	.04				
*Avoiding Confrontation*							
Overall Model				4.07	6, 146	.00	.11
Mate Availability	.03	.98	.00				
Condom Attitudes	− .78	.44	− .06				
Romantic Relational	3.53	.00	.29				
Relational	− 1.10	.28	− .09				
Fidelity	− 3.30	.00	− .26				
Appearance	.15	.88	.01				

#### Masculine Norms and Sexual Deception

Additional multiple regression analyses examined whether masculine norms subscales, with condom attitudes and mate availability, significantly predicted each subscale of sexual deception (see Table 3).

*Blatant Lying*. The model showed a significant positive association, *F*(7, 125) = 4.19, *p* < .001. However, only playboy norms and power over women emerged as significant positive predictors.

*Self-Serving Deception*. The model had a significant positive association with self-serving deception, *F*(7, 125) = 2.87, *p* < .05, with playboy norms as the only significant positive predictor.

*Avoiding Confrontation*. A significant association was found, *F*(7, 125) = 2.28, *p* < .05; however, there were no significant predictors.

#### Feminine Norms and Sexual Deception

Similarly, multiple regression analyses were conducted to determine if the feminine norms subscales, condom attitudes, and perceived mate availability were significantly associated with each sexual deception subscale (see Table 4).

*Blatant Lying*. The model showed a significant association with blatant lying *F*(6, 146) = 3.44, *p* < .001; however, increased fidelity was the only significant inverse predictor.

*Self-Serving Deception*. The model was a significant predictor, *F*(6, 146) = 3.71, *p* < .001, with more positive condom attitudes and increased fidelity indicating less deception.

*Avoiding Confrontation*. The model had a significant association with avoiding confrontation *F*(6, 146) = 4.07, *p* < .001, with increased fidelity and romantic relational norms being significant inverse predictors.

### Summary

These findings suggest that gender norms, relationship status, and condom attitudes all play a role in shaping patterns of sexual deception. In men, deception was greatly influenced by playboy norms and power dynamics. However, in women, deception varied by relationship status and adherence to certain norms. Furthermore, fidelity norms were associated with less deception, while romantic relational norms were associated with increased deception.

## Discussion

The study contributes to the empirical literature about sexual communication on HBCU campuses (see Johnson, 2017; Younge et al., 2014; Younge et al., 2018) by investigating the relationship between gender norms, perceived mate availability, condom attitudes, and sexually deceptive behaviors. The study found a significant relationship between conformity to gender norms and sexual deception among Black HBCU students, which aligns with existing literature (see Easterling et al., 2019). Additionally, this study provided data on gender and relationship status in sexual deception.

In accordance with previous research, the entire sample of participants reported being sexually deceptive to some degree (see Easterling et al., 2019; Guthrie & Kunkel, 2013). This is partially consistent with the findings and suggestions of Oswald et al. (2021), who described a cultural shift in sexual deception that is not gender specific. The nature of deception varied according to gender norms and the type of relationship. For men, relationship status did not influence deception. However, for women, the adherence to gender norms and the related deception differed depending on their relationship status. Considering SET underpinnings (see Homans, 1961; Thibaut & Kelley, 1959), the status or type of relationship might accompany a request to adhere to a specific norm (as well as being deceptive) if there is a perceived benefit or reward to being in a relationship with that person. For example, women in committed relationships demonstrated increased fidelity with less blatant lying than women not in a relationship. The latter group reported less fidelity and negative condom attitudes, but more blatant lying. While these were inverse associations, for either context, the increased action (norm or blatant lies) could be costly when seeking the type of interpersonal reward in their current relationship (commitment vs. casual).

Conversely, there could be a higher cost when these associations are positive, as women in casual relationships had increased appearance norms associated with increased self-serving deception. In this scenario, the increased adherence and deception place their partners at greater power since it would cost more to receive the intended reward. For men, relationship status was not imperative. However, the adhered norms (playboy, power over women) and deception hold equal cost since every sought partner (presumable women) would not be willing to “reward” said behaviors, considering the various relationship types women appear to be seeking. Although certain norms may be adhered to out of personal belief, findings grant theoretical utility to SET underpinnings on how norms are adopted and/or deception is used to obtain a desired relationship. More specifically, findings provide empirical merit for how students adopt norms for reward-seeking in interpersonal relationships (i.e., monogamy, number of partners) with student peers on HBCU campuses (see Johnson, 2017).

Previous literature suggests why participants sampled confirmed specific gender norms. The findings revealed that men confirmed “playboy” or “power over women” norms; however, the women sampled indicated more variety in norms contextual to relationship type. During the early phase of relationships, people often divulge specific images of themselves to reduce uncertainty from their intended partner (see Yang et al., 2014). Black HBCU students might be following certain norms associated with being college students or related to the type of relationship they are currently in or seeking. For example, women in casual relationships reported adhering more to appearance and romantic relationship norms when engaging in deception. These norms may be aligned with transitioning from a casual to a committed relationship, as committed relationships often emphasize the importance of commitment.

On the contrary, the adherence to “playboy” and “power over women” norms in young men may result from not searching for any specific type of relationship, since these norms align with certain sexual beliefs of male college students (see Eaton & Matamala, 2014; Jozkowski & Peterson, 2013). Within the HBCU environment, cultural devices (i.e., religion, hip-hop culture, media trends) further inform how specific images are transmitted interpersonally by heterosexual men and women in their romantic encounters, which has further resulted in dishonesty in retaining partners (see Chandler et al., 2021; Johnson, 2017; Ward et al., 2020). Immersion in cultural devices can influence how young Black heterosexual men and women adhere to socially prescribed norms that accompany intended interpersonal outcomes, which grants further theoretical utility to the notion that adherence is a cost for the desired reward (see Homans, 1961; Thibaut & Kelley, 1959). Future research should investigate the impact of culturally transmitted images on the behaviors of the target group, particularly in their influence on interpersonal communication and romantic partner choices.

Aligning with previous literature, condom attitudes (and presumably use) were inconsistent (see El Bcheraoui et al., 2013; Graham et al., 2016; Younge et al., 2018). Women had more positive attitudes toward condoms than men. Based on SET, attitudes toward condoms could be linked to interpersonal communication. Feelings toward condoms might affect potential partnerships, aligning with existing literature on how partner preferences shape condom attitudes (see El Bcheraoui et al., 2013). However, the inconsistency in attitudes toward condom use observed in this study and among HBCU students in the empirical literature warrants further and more specific investigation. For example, previous literature found that African American male students have difficulty discussing their sexual history and condom use (see Graham et al., 2016), while African American female students choose not to use condoms when they know their partner’s HIV status (see McLaurin-Jones et al., 2017). Future research should explore condom use and attitudes among HBCU students, especially considering the implications of condom non-use and that inconsistencies are empirically re-occurring on HBCU campuses with gendered imbalances (see Johnson, 2017).

Although perceived mate availability was not a significant predictor, consistent with prior research, men still reported a higher availability of mates on campus (Hall et al., 2014; Johnson, 2017; Stackman et al., 2016). The association between certain norms and deception may reflect the underlying gender imbalance, with additional variables. Previous findings established that mate-guarding (mate retention) behaviors often arise in contexts of limited mate availability (see Arnocky et al., 2014). However, it is unclear how these behaviors are enacted among HBCU campuses. Therefore, future research should continue to investigate mate availability within the target group. Exploring additional variables of interpersonal communication based on perceived mate availability on HBCU campuses can provide further insight into how certain norms may be enacted with deception. Further, they may speak to the theoretical possibility that adhering to these gendered norms are costly in receiving a reward or social status based on the understanding that there are not many available mates (see Homans, 1961; Thibaut & Kelley, 1959).

An additional finding revealed that as students advanced in age, they were more likely to engage in deception. This trend may reflect adaptation to a campus culture where having multiple or concurrent partners is perceived as normative (Easterling et al., 2019; Hall & Jones, 2020; Johnson, 2017; Jozkowski & Peterson, 2013; Pittman et al., 2019). However, the reasons behind increased deception remain unclear and merit further investigation. It is possible that as students age, they develop greater interpersonal or strategic communication skills, which may enable them to engage in deceptive behaviors. Alternatively, the increase may signal a growing urgency to secure romantic or sexual partners during the final stages of college. These patterns suggest potentially complex motivations underlying deception in emerging adulthood. Further research should examine how cultural elements shape interpersonal communication and sexual deception among HBCU campus students as they get older. If additional or alternate cultural norms influence these behaviors, prevention and intervention practices may be necessary for HBCU students entering a college/university that emphasizes honesty in sexual relationships while addressing socially prescribed norms associated with deceptive actions.

### Strengths and Limitations

This study adds to the understanding of sexually deceptive behaviors, gender norms, and condom attitudes of Black HBCU students; however, it is not without limitations.

One point of consideration noted is that some of the established measures were not typically utilized with Black college student/HBCU student samples. As such, the researchers understood the possibility that measures for gender norms (Parent & Moradi, 2009, 2010), and sexual deception (Marelich et al., 2008), may reflect the norms of non-Black college students from non-HBCU institutions. Therefore, the researchers aimed to establish measurement equivalence by using measures previously established for Black/HBCU student samples (see Hall et al., 2014; Boyer et al., 2008), which aligns with assertions from Bravo (2003) and Burlew et al. (2009), that cultural groups may differ in how they connect specific behaviors to traits. The measures utilized were empirically established and theoretically understood to be elements akin to Black college students on HBCU campuses (see Hall et al., 2014; Johnson, 2017; Younge et al., 2013). Further, these measures could provide a bridge of connection to the gender norm and sexual deception variables being examined. Second, the research established reliability with all measures/scales prior to data analysis. Despite these considerations and significant findings, some of the measures utilized had lower alpha scores than standard ones, possibly due to these cultural and/or racial differences between the participants sampled and those on which the scales were constructed and established, as discussed. Therefore, future research to evaluate psychometric properties may be necessary to depict certain behaviors in the targeted group accurately.

An additional point of consideration is the heteronormative and binary nature of the utilized measures, which should be noted. The established measures for gender norms arguably make these findings limited in their generalizability to those who identify as heterosexual; while this is not a holistic drawback, it does speak to the necessity for inclusivity in future research. Future research should account for differences in gender identification and sexual orientation to establish measures that extend beyond the heteronormative nature traditionally found in psychological research. Another limitation of this study is the potential biases in self-reporting. Since this study explored gender norms and sexually deceptive behaviors, it is possible that participants reported “norms” and behaviors that they aligned with what they deemed “typical” at that age. On the other hand, considering what was being asked, they may not have wanted to be honest in their reporting. Despite these limitations, this study contributes to the literature on HBCU students. Furthermore, this study unpacks how conformity to certain socially prescribed gendered norms may impact their honesty in sexual communication, which is helpful to incoming HBCU students entering college on the psychosocial implications of their actions.

## Conclusions

This research highlights the need for further attention to the interpersonal and environmental risks associated with gendered norms and sexual deception among HBCU students. Targeted interventions can foster open and comfortable dialogue regarding sexual communication while promoting authentic self-perceptions of heterosexual Black men and women against pressures from media and society (see Johnson, 2017). Recent studies show the complex and nuanced ways in which sexual deception can occur (see Oswald et al., 2021), with this paper highlighting how socially prescribed norms are used in sexual communication. Given that HBCU students may adopt said norms due to prevalent media and cultural imagery that shapes their interpersonal behaviors (see Johnson, 2017; Chandler et al., 2021; Ward et al., 2020), it is essential to examine these influences to mitigate the potential adverse effects. Additionally, continued exploration of these norms and their broader impact on interpersonal communication within the HBCU environment is critical for strengthening campus-wide efforts to foster healthier interpersonal dynamics.
